# Space Environmental Factor Impacts upon Murine Colon Microbiota and Mucosal Homeostasis

**DOI:** 10.1371/journal.pone.0125792

**Published:** 2015-06-17

**Authors:** Lauren E. Ritchie, Stella S. Taddeo, Brad R. Weeks, Florence Lima, Susan A. Bloomfield, M. Andrea Azcarate-Peril, Sara R. Zwart, Scott M. Smith, Nancy D. Turner

**Affiliations:** 1 Intercollegiate Faculty of Genetics, Texas A&M University, College Station, Texas, United States of America; 2 Nutrition & Food Science Department, Texas A&M University, College Station, Texas, United States of America; 3 Department of Veterinary Pathobiology, Texas A&M University, College Station, Texas, United States of America; 4 Division of Nephrology, Department of Medicine, University of Kentucky, Lexington, Kentucky, United States of America; 5 Department of Health and Kinesiology, Texas A&M University, College Station, Texas, United States of America; 6 Department of Cell Biology and Physiology, University of North Carolina School of Medicine, Chapel Hill, North Carolina, United States of America; 7 Human Health and Performance Directorate, NASA Lyndon B. Johnson Space Center, Houston, Texas, United States of America; Colorado State University, UNITED STATES

## Abstract

Astronaut intestinal health may be impacted by microgravity, radiation, and diet. The aim of this study was to characterize how high and low linear energy transfer (LET) radiation, microgravity, and elevated dietary iron affect colon microbiota (determined by 16S rDNA pyrosequencing) and colon function. Three independent experiments were conducted to achieve these goals: 1) fractionated low LET γ radiation (^137^Cs, 3 Gy, RAD), high Fe diet (IRON) (650 mg/kg diet), and a combination of low LET γ radiation and high Fe diet (IRON+RAD) in male Sprague-Dawley rats; 2) high LET ^38^Si particle exposure (0.050 Gy), 1/6 G partial weight bearing (PWB), and a combination of high LET^38^Si particle exposure and PWB in female BalbC/ByJ mice; and 3) 13 d spaceflight in female C57BL/6 mice. Low LET radiation, IRON and spaceflight increased *Bacteroidetes* and decreased *Firmicutes*. RAD and IRON+RAD increased *Lactobacillales* and lowered *Clostridiales* compared to the control (CON) and IRON treatments. Low LET radiation, IRON, and spaceflight did not significantly affect diversity or richness, or elevate pathogenic genera. Spaceflight increased *Clostridiales* and decreased *Lactobacillales*, and similar trends were observed in the experiment using a ground-based model of microgravity, suggesting altered gravity may affect colonic microbiota. Although we noted no differences in colon epithelial injury or inflammation, spaceflight elevated TGFβ gene expression. Microbiota and mucosal characterization in these models is a first step in understanding the impact of the space environment on intestinal health.

## Introduction

Spaceflight exposes astronauts to environmental inputs that are different from those on earth including microgravity and radiation exposure. NASA is concerned with the health risks associated with these environmental factors, particularly radiation, as they have been shown to have deleterious effects such as increasing the risk of heart disease, cataracts, and accelerated bone loss [1–3]. The limited food system on board spacecraft can further exacerbate these environmental challenges by providing relatively high iron intakes, which have historically exceeded recommended intakes by 3–4 fold [4].

Linear energy transfer (LET), or the amount of energy that is transferred to a substance as the radiation passes through, impacts the extent of radiation damage to biological systems [5]. Low LET radiation, such as gamma and x-rays, is more likely to have indirect effects on cells through the generation of free radicals (e.g., H•, •OH, H_2_O_2_, and *e* ^-^_aq_), while high LET radiation can physically damage DNA and proteins [5, 6]. Both high and low LET radiation exposure can compromise plasma and organelle membrane integrity and cause DNA damage and DNA methylation [5, 7]. Once a cell becomes irradiated, possible outcomes include genomic mutations, cell cycle arrest, and transformation to a potentially carcinogenic cell [7, 8].

Reactive oxygen species are generated within cells immediately after exposure to radiation and could be exacerbated by other environmental factors, including nutrient status. For example, oxidative stress can be propagated in the intestine by consumption of a diet containing pro-oxidants such as iron, which increases the risk of forming cytotoxic compounds and free radicals [9]. Physiological outcomes induced by high iron include intestinal barrier damage, hyperproliferation, downregulated repair mechanisms, as well as altered microbiota and luminal concentration of short chain fatty acids (SCFA) [10, 11].

Very little research has been done to understand the effect of low dose ionizing radiation (<1 Gy) and microgravity on the colon luminal environment and intestinal epithelium function. Large radiation doses (>8 Gy) can cause detrimental effects such as direct mucosal toxicity, delayed gastric emptying and secretion, and villous shortening [7, 12]. Some data suggests that radiation exposure can affect the intestinal microbiota, and the presence and composition of these commensal bacterial groups has an effect on radiation induced-lethality [13–15]. Furthermore, we have reported that consumption of a diet with elevated dietary iron, by itself and in combination with low LET radiation, can alter the concentration of fecal SCFA [16], suggesting alterations to the composition of the intestinal microbiota or a functional change in microbial metabolism. An understanding of how the aforementioned environmental insults affect the microbiota is of utmost importance, as alterations to commensal bacterial groups have been implicated in the onset and recurrence of chronic intestinal inflammation and gastrointestinal diseases [17, 18].

Mitigating the effects of oxidative stress caused by a combination of radiation exposure and elevated iron status is an important area of research, as no countermeasures have been thoroughly studied. Results from this research would benefit not only astronauts, but others exposed to radiation during medical diagnostic tests, and occupational radiation exposures. To our knowledge, no study has characterized the effects of fractionated, low doses (0.5–3 Gy) of low LET ionizing radiation with a high dietary iron load, or one dose high LET ionizing radiation with simulated lunar gravity conditions on colon microbiota and mucosal homeostasis. We hypothesized that characteristics of the space environment (i.e., diet, radiation, and altered gravity) would alter the intestinal microbiota and epithelium. Therefore the aims of the independent studies summarized in this work were to characterize the effects of: 1) low LET radiation exposure and high Fe diet, 2) partial weight bearing (1/6 G) and high LET radiation exposure, and 3) short term spaceflight, on the intestinal microbiota, mucosal expression of gene targets associated with microbial signaling and epithelial repair, and measurements of colonic injury and inflammation in rodents.

## Materials and Methods

### Animals and diets

The experiments outlined below describe three independent studies. Experimental design was optimized for each study individually in order to execute funded experiments, which were not associated with this body of work. Thus, animal model, control diet, and housing continuity across experiments was not possible. Furthermore, funding for methodologies described herein was limited and dictated scope of analysis.

#### Experiment 1—fractionated low LET gamma radiation and high dietary iron in rats

The aim of Experiment 1 was to characterize the impacts of fractionated doses of low LET radiation exposure and high dietary iron on fecal bacterial populations and colon homeostasis. Thirty two, 12-week-old, male Sprague-Dawley rats (Charles River Laboratories, Wilmington, MA, USA) were acclimated to an adequate iron diet (45 mg iron (ferric citrate)/kg diet) for 3 weeks (AIN-93G; Research Diets, New Brunswick, NJ, USA) and then assigned to one of four groups: adequate Fe diet/no radiation (CON), adequate Fe diet/radiation (RAD), high Fe diet (650 mg iron (ferric citrate)/kg diet)/no radiation (IRON), and high Fe diet/radiation (IRON+RAD). Animals received the assigned diet for 4 weeks. On day 14 of the experiment, animals began exposures to a 0.375 Gy fractionated radiation dose of ^137^Cs every other day for 16 days (3 Gy total dose) at the NASA Johnson Space Center. Animals in both the sham and radiation groups were placed in a restraint tube (Battelle, Geneva, Switzerland) for about 10 min per treatment session. On day 29 (24 h after last radiation exposure), animals were euthanized using isoflurane vapor. The colon was resected, feces removed for microbial sequencing analysis, and colonic mucosa scraped in order to determine gene expression. Experiments outlined in this work are an extension of a previous study, which analyzed the impact of diet and radiation on the colon epithelium and SCFA concentrations in the feces and have been reported previously [16].

#### Experiment 2—high LET 28silicon particle exposure and partial weight bearing (1/6 G) in mice

The aims of Experiment 2 were to determine the effect of high LET radiation and partial/lunar gravity (1/6 G) on: 1) fecal bacterial populations, and 2) expression of genes involved in microbial signaling and tissue repair.

Because of sample and funding limitations in this experiment, an aliquot of extracted bacterial DNA was taken from each animal and combined into a composite sample for each experimental group. The isolated composite DNA samples were used for pyrosequencing and further phylogenetic analysis in order to perform an initial and exploratory characterization of the impact of high LET radiation and unloading on microbiota. A description of the methodologies for this experiment can be found in S1 Appendix.

#### Experiment 3—combined high LET and low LET radiation exposures and microgravity conditions during short term space flight in mice

The aims of Experiment 3 were to: 1) characterize fecal bacterial populations, 2) determine expression of genes involved in microbial signaling and tissue repair, and 3) assess colonic epithelial injury and inflammation in mice after short-term spaceflight.

Fourteen, 49-day-old, female C57BL/6 mice (Charles River, Raleigh, NC, USA) were acclimated to NASA Rodent Foodbar diets and water for 2 wk [19]. All animals were then loaded into NASA-designed animal enclosure modules (AEM’s) and given *ad libitum* access to food and water for the remainder of the study. Half of the animals were flown on the Space Shuttle Atlantis mission STS-135 for a period of 13 days. Lights in both the ground and flight AEM were on a 12/12 h light/dark cycle. Following landing (3–7 hours), all animals were anesthetized by isoflurane gas and euthanized by decapitation. The colon was resected, feces removed, a 1 cm section of the colon was fixed for histological preparations, and the remainder of the colon mucosa was harvested by scraping as part of a tissue sharing program.

### Sample collection and processing

#### Scraped mucosa

After fecal material was removed, the colon was washed twice in RNase free Phosphate-Buffered Saline (PBS) and scraped on a chilled RNase free surface.

Experiment 1. Mucosa was transferred to an RNase free homogenization tube along with 500 μl of Denaturation Solution (Ambion, Austin, TX), homogenized by pipetting 6–7 times, snap frozen, then transported to Texas A&M University on dry ice and stored at -80°C.

Experiment 3. Mucosa was transferred to an RNase free homogenization tube along with 500 μl of RNA*later* (Ambion, Austin, TX), snap frozen, then transported to Texas A&M University on dry ice and stored at -80°C.

#### Feces collection and microbial DNA isolation

Upon resection of the colon at termination, fresh fecal samples were collected and transferred to sterile cryotubes, snap frozen, then transported to Texas A&M University on dry ice and stored at -80°C. DNA was isolated from homogenized fecal samples using a FastDNA SPIN kit according to the manufacturer’s instructions (MP Biomedicals, Solon, OH) [20]. This method includes a physical disruption step to ensure genomic DNA isolation from Gram positive bacteria. Purified DNA was stored at -80°C. A negative control containing H_2_O instead of sample was purified in parallel to each set of extractions to screen for contamination of extraction reagents.

Bacterial DNA isolated from each sample collected in Experiments 1 and 3 was used for pyrosequencing and further phylogenetic analysis (described below). For reasons previously described, composite samples generated for each treatment in Experiment 2 were used for these analyses.

### Inflammation and injury histological scores

In Experiment 3, a 1 cm segment was removed from the distal end of the colon and fixed in 70% EtOH solution prior to embedding in paraffin. Paraffin embedded sections were stained with hematoxylin and eosin (H&E), and the degree of inflammation and morphological injury induced by space flight was assessed by a board-certified pathologist in a blinded manner. The degrees of inflammation (score of 0–3) and epithelial injury (score of 0–3) in microscopic cross sections of the colon were graded as described previously [21].

### Measurement of gene expression using real-time PCR

Total RNA was isolated from scraped mucosal samples using Phase Lock Gel tubes (5 Prime, Gaithersburg, MD) and the ToTALLY RNA Kit or RNAqueous kit (Ambion, Austin, TX) followed by DNase treatment (DNA-*free* Kit, Ambion, Austin, TX). RNA quality was assessed using an Agilent Bioanalyzer. First strand cDNA was synthesized using random hexamers, oligo dT primer (Promega, Madison, WI), and Superscript III Reverse Transcriptase following manufacturer’s instructions (Invitrogen, Carlsbad, CA).

Real-time PCR was performed on select genes involved in the TLR signaling cascade (i.e., TLR2, TLR4, TLR9, MyD88, NFκB, TNFα, IL-6, IL-1b), short chain fatty acid transport (i.e., Slc16a1, Slc5a8), and epithelial barrier restitution (i.e., TGFβ, TFF3) using Taqman Array Plates (Applied Biosystems, Foster City, CA) and a ABI 7900 HT thermocycler (Applied Biosystems, Foster City, CA) (S1 Table). To screen for potential contamination of PCR reagents, a negative PCR control of H_2_O instead of cDNA template was used. Expression levels were normalized to 18S gene expression.

### 16S rRNA bacterial tag-encoded FLX amplicon pyrosequencing

16S amplicon sequencing was performed as previously described [22]. Briefly, initial amplification of the V1-V2 region of the bacterial 16S rDNA was performed on total DNA isolated from fecal samples. Master mixes for these reactions used the Qiagen Hotstar Hi-Fidelity Polymerase Kit (Qiagen, Valencia CA) with a forward primer composed of the Roche Titanium Fusion Primer A (5’-CCATCTCATCCCTGCGTGTCTCCGACTCAG -3’), a 10 bp Multiplex Identifier (MID) sequence (Roche, Indianapolis, IN) unique to each of the samples, and the universal bacteria primer 8F (5'-AGAGTTTGATCCTGGCTCAG-3') [23]. The reverse primer was composed of the Roche Titanium Primer B (5’-CCTATCCCCTGTGTGCCTTGGCAGTCTCAG -3’), the identical 10 bp MID sequence as the forward primer and the reverse bacteria primer 338R (5’-GCTGCCTCCCGTAGGAGT-3’) [24] which span the V1-V2 hypervariable region of the bacterial 16S rDNA [24]. The thermal profile for the amplification of each sample had an initial denaturing step at 94°C for 5 minutes, followed by a cycling of denaturing of 94°C for 45 seconds, annealing at 50°C for 30 seconds and a 90 second extension at 72°C (35 cycles), a 10 minute extension at 72°C and a final hold at 4°C. Each sample was individually gel purified using the E-Gel Electrophoresis System (Life Technologies, Invitrogen). To ensure equal representation of each sample in the sequencing run, each barcoded sample was standardized by calculating equimolar amounts prior to pooling. Pooled samples of the 16S rDNA multiplexed amplicons were sequenced on a Roche 454 Genome Sequencer FLX Titanium instrument (Microbiome Core Facility, Chapel Hill NC) using the GS FLX Titanium XLR70 sequencing reagents and protocols.

### Amplicon sequencing data analysis

Analysis of amplicon sequencing data was carried out using the QIIME pipeline [25]. The combined raw sequencing data plus metadata describing the samples were de-multiplexed and filtered. Next, data were denoised using Denoiser software as described previously [26]. Sequences were grouped into OTUs (Operational Taxonomic Units) at a 97% level to approximate species-level phylotypes using Uclust [27]. OTU sequences were aligned and OTU tables containing the counts of each OTU in each sample were used to calculate mean species diversity of each sample (alpha diversity) and the differentiation among samples (beta diversity). Alpha and beta diversity measures were used to calculate a Chao species richness estimate and a Shannon Weaver diversity index for each sample [28, 29]. To evaluate the similarities between bacterial communities a combination of Unifrac significance and principal coordinate analysis (PCoA) using Fast Unifrac [30] and network analysis were performed to compare samples based on sample time and treatment.

The 16S rRNA gene sequences obtained were deposited into NCBI’s Sequence Read Archive (SRA) database with accession number GSE68738.

### Statistical analysis

Data were analyzed using two-way analysis of variance (ANOVA) or one sample t-test (TTEST) in SAS 9.1 (SAS Institute, Inc.). Differences detected by pairwise comparisons with *P*-values <0.05 are indicated in each table by superscripts (a, b, c). Data sets from Experiment 3 that were not normally distributed were analyzed using Kruskal-Wallis test. On completion of all analyses, the uncorrected *P*-values underwent a single multiple-testing correction procedure using the Benjamini and Hochberg false discovery rate (FDR) method. Q values are reported for results remaining significant after adjusting for multiple comparisons (0.2 threshold). Within each table, pairwise comparisons with a q value <0.2 are indicated with an asterisk.

## Results

### Experiment 1—Effects of low LET gamma radiation exposure and high dietary iron on the intestinal microbiota in rats

**Fig 1:**
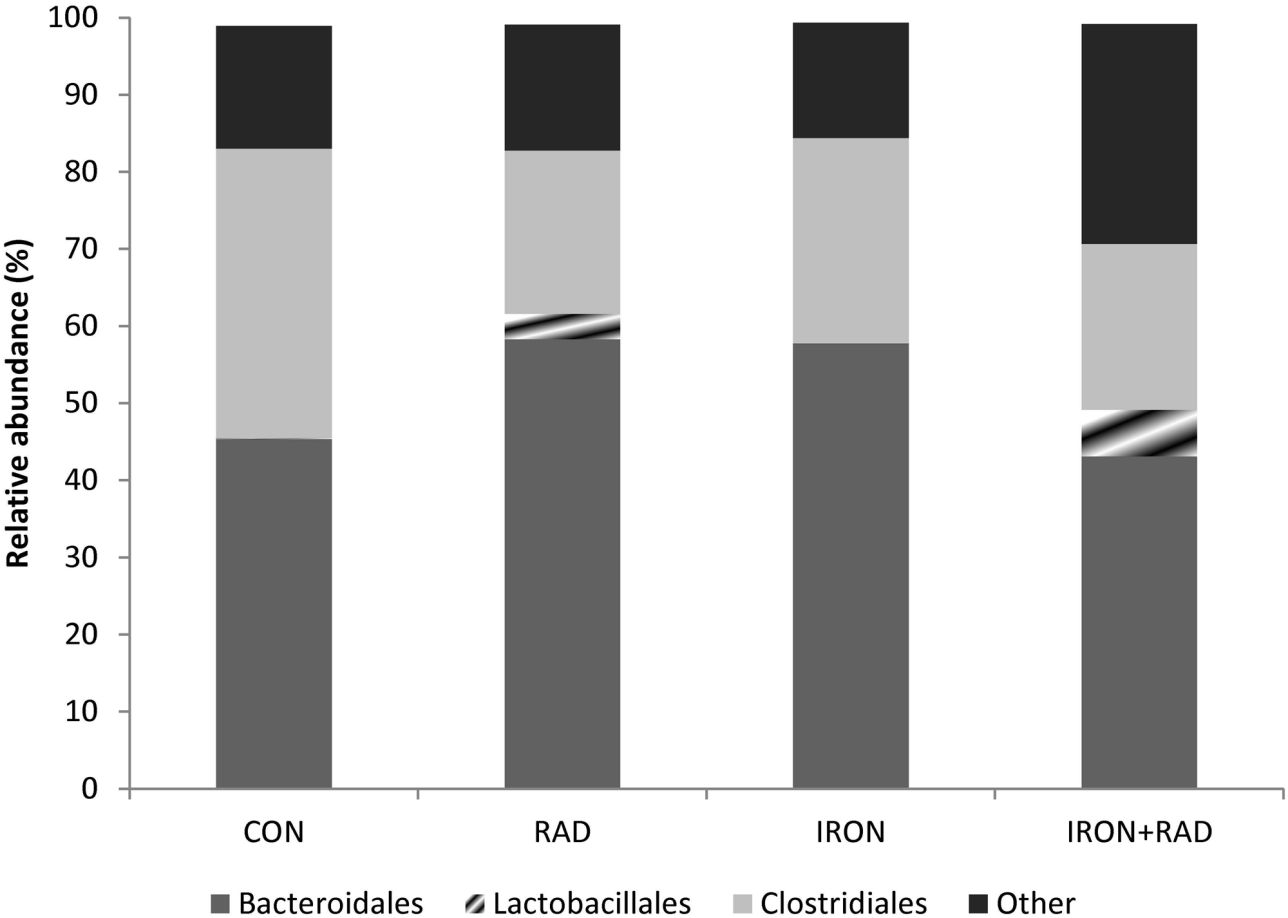
Relative abundance (%) of bacterial taxa at the order level in the feces of rats resulting from exposure to high dietary iron or low LET radiation (Experiment 1). Actual values in S2 Table.

We observed a significant radiation effect on the *Clostridiales* order (q = 0.17, S2 Table), with CON having a significantly higher proportion of *Clostridiales* compared to RAD and IRON+RAD (both q = 0.14, Fig 1). IRON+RAD had a significantly higher abundance of Unknown OTUs within the *Bacteroidetes* phylum compared to CON, RAD, and IRON groups (q<0.2, S2 Table). We observed no significant differences in species diversity (Shannon Weaver index) in response to dietary iron level, irradiation or their interaction (Table 1). Although not statistically significant, species richness (Chao index) was numerically higher in IRON+RAD rats compared to IRON rats (Table 1). Additionally, we observed relatively large changes in the following phylogenetic taxa that did not reach significance after adjusting for multiple comparisons. The abundance of *Firmicutes* was lower in the RAD group compared to CON (S2 Table). IRON rats tended to have an increased proportion of *Bacteroidetes* (17%) and decreased proportion of *Firmicutes* (31%) compared to CON. RAD elevated *Lactobacillales* with IRON+RAD having a higher proportion compared to CON and IRON.

**Table 1:** Chao and Shannon Weaver indices for microbial populations in the feces of rats and mice exposed to various space-relevant environmental conditions.^1^

	Experiment 1				Experiment 3	
	CON (n = 8)	RAD (n = 8)	IRON (n = 8)	IRON+RAD (n = 8)	Ground (n = 7)	Flight (n = 7)
Shannon Weaver	2.70 ± 0.11^a^	2.62 ± 0.23^a^	2.67 ± 0.23^a^	3.12 ± 0.32^a^	4.46 ± 0.27^a^	4.04 ± 0.27^a^
Chao	68.19 ± 2.49^ab^	67.22 ± 5.15^ab^	62.70 ± 4.24^a^	80.44 ± 8.63^b^	84.81 ± 4.74^a^	82.95 ± 14.01^a^

^1^Data are LS mean±SEM.

Means differing after initial analysis are indicated by different superscript letters and the remaining significant pairwise comparisons after performing an FDR procedure are indicated by * (q<0.2).

CON = control iron diet/sham radiation, RAD = control iron diet/low LET γ radiation, IRON = high iron diet/sham radiation, IRON+RAD = high iron diet/low LET γ radiation.

### Experiment 2—Effect of high LET ^28^Si particle exposure and PWB on mucosal gene expression and the intestinal microbiota in mice

The outcomes from this preliminary experiment, which measured the effect of high LET radiation exposure alone or in combination with unloading using a validated ground-based model of microgravity, did not provide an opportunity to detect statistical differences in bacterial populations among treatment groups, as composite samples were used for analysis. However, there were relatively large changes in the proportion of some bacterial orders detected with unloading (S1 Appendix, S3 Table). We did not observe a significant effect of radiation or partial weight-bearing on relative expression of any gene targets analyzed (S1 Table).

### Effect of spaceflight on colon morphology, mucosal gene expression, and intestinal microbiota in mice (Experiment 3)

#### Body weight and experimental diet intake

**Fig 2:**
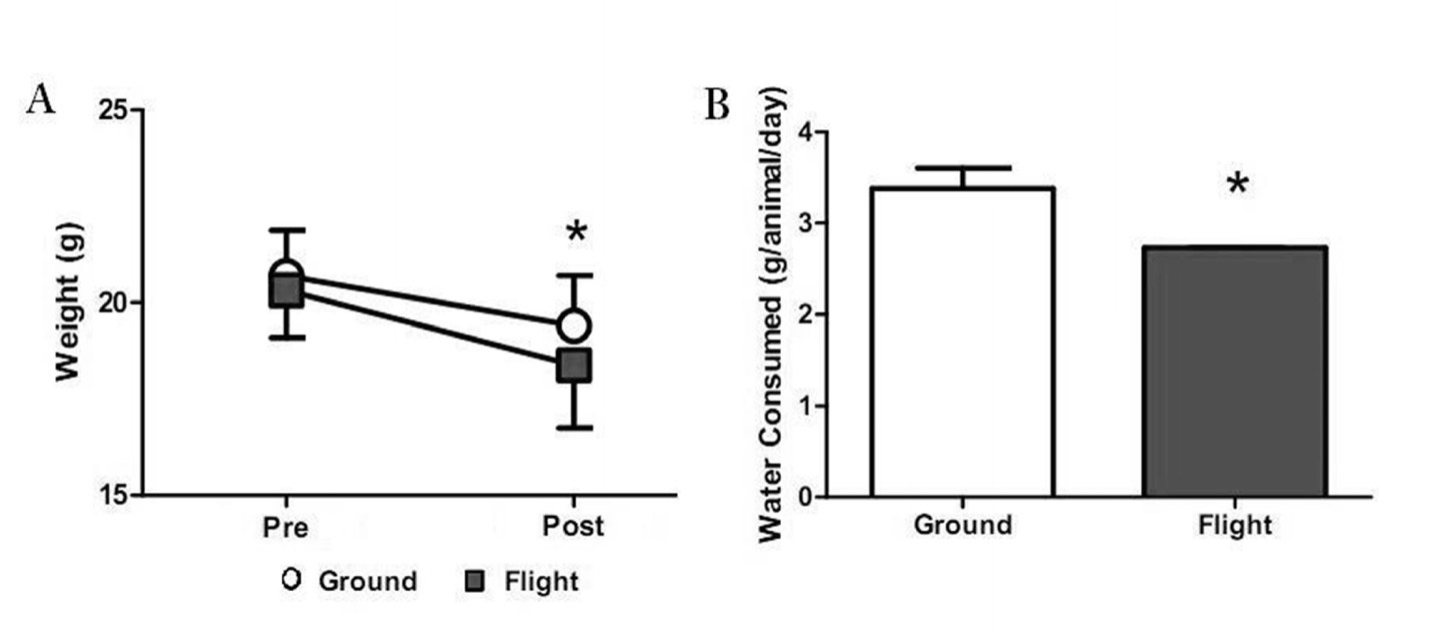
A) Mean body weight was significantly lower in flight mice compared to ground controls post-flight (p<0.05). B) Total water consumption was significantly lower in flight mice compared to ground controls. Means with * differ between ground and flight mice (p<0.05). Data are LS means ±SEM.

Post flight, we observed significantly lower body weights in flight mice compared to ground controls (Fig 2A). Food intake was not significantly different between groups (not shown), however, water intake during flight was significantly suppressed in flight mice compared to ground mice (Fig 2B). STS-135 radiation dosimetry revealed that the animals were exposed to a maximum measured dose of 5.46 ± 0.07 mGy, consisting of 112.6 μG/d galactic cosmic rays and 117.8 μGy/d trapped particles.

#### Microbial diversity, species richness, and taxonomic structure analysis

**Fig 3:**
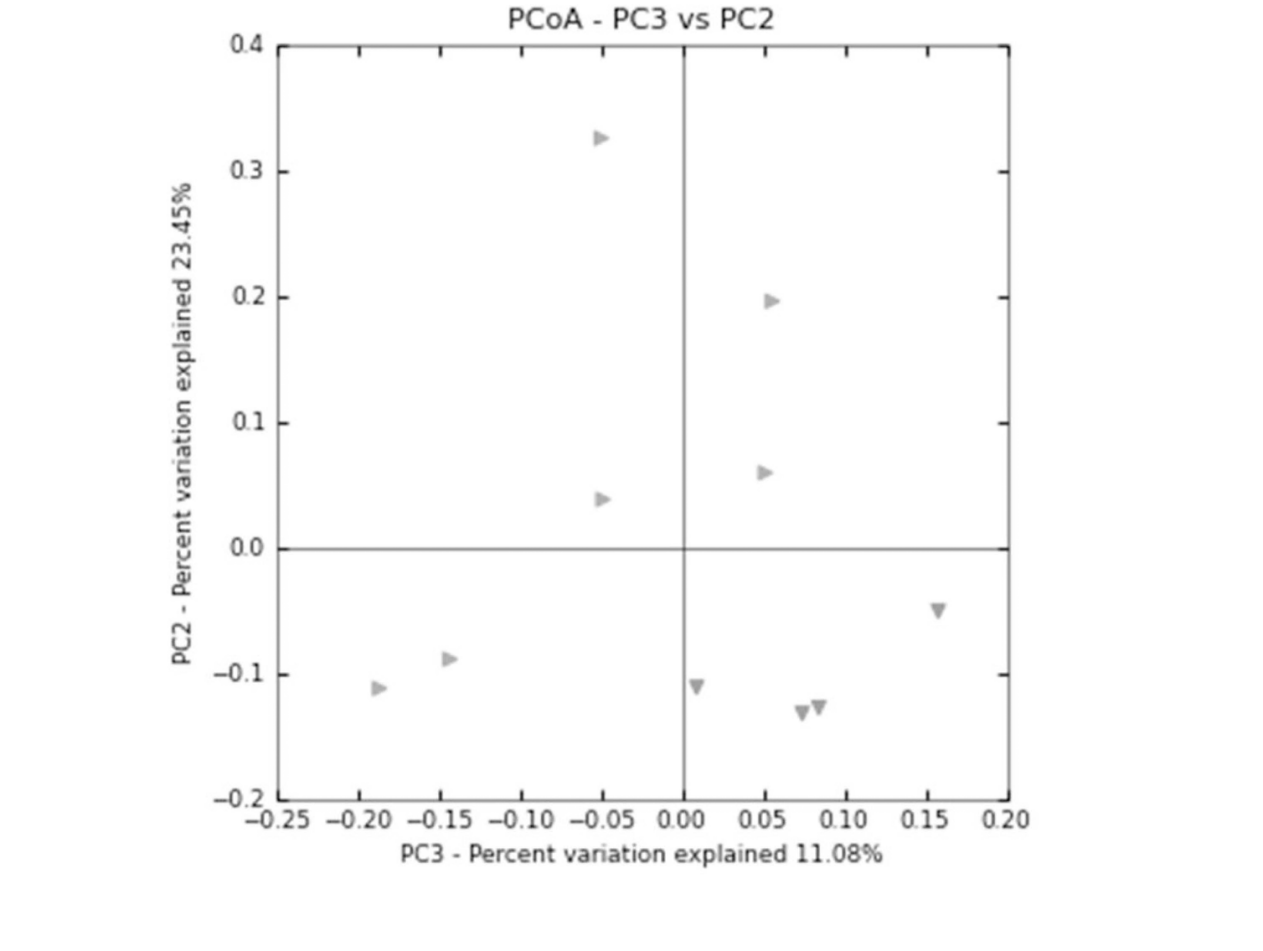
Principal coordinate analysis (PCoA) plot of samples from ground and flight mice. Flight mice data points clustered relatively tightly within the bottom right quadrant, whereas ground controls are distributed throughout the remainder of the plot. The PCoA analysis illustrates the differences in bacterial populations in the feces of flight mice compared to ground controls.

**Fig 4:**
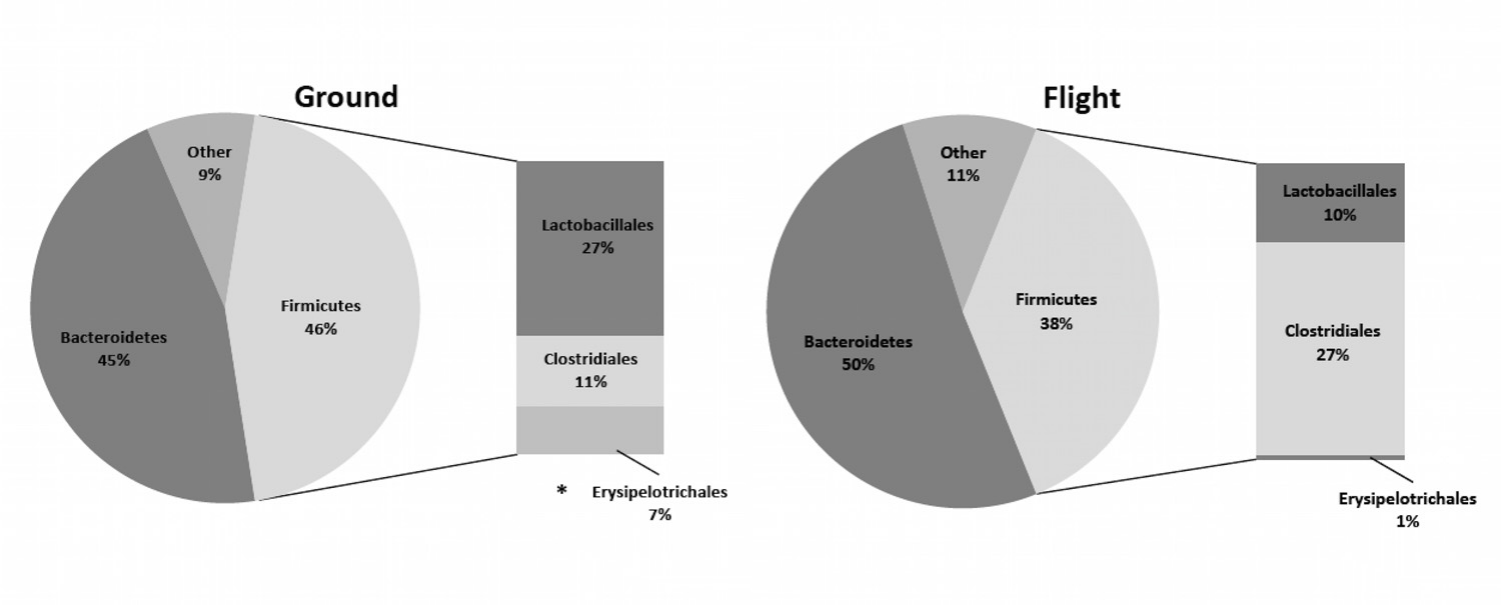
Relative abundance (%) of bacterial taxa in feces from ground versus flight mice. Taxa with * differ between ground and flight mice (p<0.05). See S4 Table for actual values.

We observed a tendency for reduced species richness and diversity in flight mice relative to the ground control mice, these differences were not significant (Table 1). Unifrac and principal coordinate analysis (PCoA) of all sequences for each sample, represented as a discrete data point without overlap, revealed distinct clustering of samples collected from ground and flight mice (Fig 3), yet we observed no significant differences in OTUs at the phylum level (S4 Table). Flight mice had a numerically higher proportion *Bacteroidetes* (8%) and lower proportion of *Firmicutes* (14%) compared to ground mice (Fig 4). Ground mice had a numerically higher proportion of *Erysipelotrichales* compared to flight mice. Flight mice had a numerically higher proportion of *Clostridiales* (60%) and numerically lower proportion of *Lactobacillales* (62%) compared to ground mice (Fig 4).

#### Mucosal gene expression

We observed no significant differences in any gene targets analyzed. However, relative gene expression tended to be higher in TGFβ, TLR2, IL-6 and Slc16a1 in flight mice compared to ground control mice (S1 Table).

#### Immunohistochemical analysis of distal colon

To understand the effects of spaceflight (i.e., combined weightlessness and mixed radiation exposure) on intestinal injury and inflammation, histological sections of the distal colon were analyzed by a board certified pathologist. When comparing flight and ground mice, we observed no significant differences in intestinal injury (0.5 and 0.95 scores, respectively) and inflammatory infiltration (1.42 and 1.34 scores, respectively) as a result of short term exposures to the space environment.

## Discussion

Astronauts are exposed to different types of radiation composed of varying magnitudes of LET (protons, alpha particles (hydrogen), and a small percentage of heavy ions (e.g., Fe and Si) and electrons), which can penetrate shielding and create secondary particles [7]. Radiation exposure and other factors experienced during space flight such as diets high in iron and microgravity can elevate oxidative stress in the body, which has been shown to upregulate an immune response and is a factor in bone demineralization, muscle atrophy, cardiovascular deconditioning and cataracts [31–33]. Recent studies have shown that the intestinal microbiota can be altered by oxidative stress and have been implicated in gastrointestinal diseases like inflammatory bowel disease (IBD) and cardiovascular disease [18, 34–36]. Furthermore, our recent studies demonstrated that fecal concentrations of SCFAs are affected by low LET radiation exposure and high dietary iron, suggesting alterations to the microbiota or microbial metabolism [16]. The initiation and progression of intestinal inflammation is of upmost importance to astronaut health, as it known to increase the risk of colorectal cancers [37, 38]. Additionally, the effect of radiation on the body has been identified by NASA as one of the most significant risks associated with human space flight, and their recent reports estimated that the “probability of causation” for colon cancer is 3^rd^ highest in radiation induced cancers following galactic cosmic radiation exposure [39, 40].

Understanding how different radiation energies affect the intestinal tract is important, as previous studies have observed differences in cell survival, DNA repair, epithelial cell proliferation, and tumor incidence following low and high LET radiation exposure [41, 42]. Our study utilized three independent experiments to characterize how high and low LET radiation exposures, high iron diet, and weightlessness may affect the fecal microbiota and mucosal gene expression. Ideally each experiment would have a unified study design, as murine genotype (not gender) have been reported to affect the microbiota [43, 44]; however, accessibility to irradiated samples and minimal funding for additional experiments precluded this option. Furthermore, it is important to elucidate the combined effects of radiation exposure and microgravity on the colonic environment, as recent studies report a synergistic effect between these exposures and miRNA expression, immune activation, and bone loss [45–47]. Very little is known about how the space environment effects intestinal homeostasis; thus, the aim of this study was to characterize the effect of high and low LET radiation exposure, microgravity, and elevated dietary iron on the intestinal microbiota and gene targets associated with microbial derived signaling, metabolite transport, and epithelial barrier restitution.

Phylogenetic classification of fecal intestinal bacterial populations at the phylum level revealed similarities across the various treatments (i.e., low LET radiation, microgravity, elevated Fe diet). Following either low LET radiation exposure (RAD) or consumption of a high iron diet (IRON), we observed an increased proportion of *Bacteroidetes* and decreased *Firmicutes* compared to CON. We observed increased *Bacteroidetes* and decreased *Firmicutes* following high dietary iron exposure; however, we did not observe a synergistic effect of low LET radiation exposure and high dietary iron (Experiment 1). Similarly, animals exposed to 13 days of space flight had an increased proportion of *Bacteroidetes* and decreased *Firmicutes* compared to ground controls. This suggested that mixed radiation exposure associated with space flight (112.6 μG/d galactic cosmic rays and 117.8 μGy/d trapped particles), comprised of both high and low LET radiations produced similar effects to the predominant phyla as treatment with only one type of radiation (i.e., X ray or high charge (Z) energy (E) silicon nuclei (HZE)). These global observations of the predominant bacterial groups are of interest, as dysbiosis to these phyla have been linked to other sources of elevated oxidative stress in the body such as obesity and IBD [48, 49]. Interestingly, increased proportions of *Firmicutes* and decreased *Bacteroidetes* have been reported in those studies, which contrast our observations following radiation exposure, microgravity, and an elevated iron diet.

Historically, studies have shown that the presence of commensal bacterial populations increased radiation induced lethality in conventionalized (i.e., presence of microbiota) compared to germ free animals [13], but a direct mechanism was not elucidated. More recent studies report alterations to the microbiota after low LET X ray exposure (5–22 Gy), and observed distinct differences based on dose, amount of time following exposure, as well as presence of clinical signs such as radiation-induced diarrhea [14, 50]. One small pilot study used denaturing gradient gel electrophoresis (DGGE) to identify changes in the bacterial diversity of patients suffering from acute post-radiotherapy diarrhea (exposures to 4.3–5.4 Gy) [14]. They observed that the microbial profiles of the individuals studied (i.e., no radiation, radiation with no diarrhea, and radiation with diarrhea) clustered in unique sets, suggesting that specific bacterial populations could be associated with risk or protection after radiation exposure [14]. Another *in vivo* study demonstrated hierarchical clustering of bacterial populations from samples collected 0, 4, 11, and 21 days post 10 Gy low LET radiation exposure suggested distinct differences in the bacterial populations over time [50]. Although these studies suggest an effect of radiation on microbiota, these studies used large doses of low LET radiation exposures that are markedly higher than those used in this study (5–22 Gy compared to 3 Gy, respectively), and did not study the effects of high LET radiation exposure.

Another contradiction to the results in this study is the report of elevated proportions of pathogenic bacteria in patients with IBD and following low LET X-ray exposure at various doses [34, 50, 51]. Both insults indicated an increase in bacterial taxa (i.e., *Enterobacteriaceae*, *Escherichia coli*, *Salmonella* spp.) classified in the *Proteobacteria* phylum, which is a common constituent of the commensal intestinal flora but also contains numerous pathogenic species such as *Helicobacter*, *Klebsiella* and *Shigella*. We observed ≤0.07% *Proteobacteria* in all experimental groups analyzed in this study, which is in contrast to previous animal studies reporting an elevation in this phylum following 10 and 18 Gy X-ray exposure [50]. This suggests that modulation of this bacterial group by radiation exposure might be dose dependent. This observation is positive, as the virulence of numerous pathogens, including *Escherichia coli* and *Salmonella* and *Bacillus* spp., have been reported to be increased following simulated microgravity and space flight [52–54]. Furthermore, it is thought that microgravity has an effect on fighting bacterial infections, as studies have reported that hind limb unloaded (HLU) animals have suppressed immune system function and increased lethality when exposed to pathogenic bacteria (e.g., *Klebsiella pneumonia* and *Pseudomonas aeruginosa*) [55, 56]. Additionally, it was reported that HLU mice had elevated bacterial organ load following administration of *Klebsiella pneumonia* compared to weight bearing controls, suggesting susceptibility to bacterial translocation from the bowel [55].

To better understand the implications of the observed altered proportions of *Firmicutes* and *Bacteroidetes*, we performed further phylogenetic classification at the order level. Within the *Firmicutes* phylum we observed differences in *Lactobacillales* and *Clostridiales* in animals exposed to either low LET radiation or microgravity. Animals exposed to 13 days space flight had an increased proportion of *Clostridiales* and decreased proportion of *Lactobacillales* compared to ground controls. We observe a similar trend in the exploratory characterizations from Experiment 2, which was independent of radiation exposure, as both LUN SHAM and LUN RAD showed these population shifts. These reports are similar to those observed in patients with IBD, as it has been documented in numerous studies a suppression in lactic acid bacteria (LAB) species as well as increased *Clostridium* spp (found in *Lactobacillales* and *Clostridiales* order, respectively) [57, 58]. Results from Experiment 1 contrast these observations, as RAD and IRON+RAD animals have numerically higher proportions of *Lactobacillales* and significantly lower proportion of *Clostridiales* compared to sham irradiated animals (i.e., CON & IRON). Lower proportions of *Clostridiales* in these animals may explain our previous observation of suppressed fecal butyrate [16], since this order harbors bacterial species known to have the capability to produce this metabolite [59]. An increase in LAB is generally thought to be positive, as these bacterial populations have been shown to provide health benefits to the host (e.g., produce antimicrobial substances, compete with pathogens for epithelial binding sites) and improves symptoms of chronic intestinal inflammation [60, 61]. However, an increase in luminal H_2_O_2_ produced by these bacteria could exacerbate the effects of an elevated iron diet by creating highly reactive hydroxyl radicals through iron-mediated Fenton chemistry [9]. Although this was not directly measured in this study, increased luminal and epithelial reactive oxygen species (ROS) could potentiate local oxidative stress.

Enhanced oxidative stress has the potential to cause cellular damage and induce an inflammatory response in the colon, and we previously demonstrated that myeloperoxidase activity (a marker of neutrophil infiltration) is elevated and TLR gene expression altered in colonic mucosa by an elevated iron diet and low LET irradiation [16]. Microbial-derived signaling through pattern-recognition receptors affects gene expression, transepithelial electrical resistance, colonocyte proliferation in normal tissues [62], and serves a pivotal role in maintaining homeostasis between commensal microbiota and the host immune system [63]. Previous studies have shown that high LET radiation exposure and microgravity has a direct effect on gene expression *in vitro* and in muscle [45, 64, 65], but to our knowledge no analysis has been published describing these effects in the intestinal tract. Therefore to understand the implications of mixed radiation exposure and spaceflight on these processes, we analyzed mucosal gene expression of gene targets associated with the TLR signaling cascade, SCFA transport, and epithelial barrier restitution in Experiment 3.

We do not observe a significant effect of spaceflight for any targets associated with the TLR signaling cascade or SCFA transport. Zhou et al. reported that HLU acts synergistically with radiation exposure to markedly elevate circulating IFNα, IL-6, and TNFα in mice [66]. Although we did not analyze cytokine concentrations in plasma or other organs, we do not observe an increase in the relative expression of IL-6, IL-1b, COX-2 or TNFα in the colonic mucosa of flight mice. Additionally, immunohistological assessment of distal colon did not reveal an increase in inflammatory cells or neutrophil infiltration following spaceflight. Differences in these observations could be due to the amount of time following radiation exposure and tissue collection, or the effect of low dose gamma irradiation compared to mixed radiation exposure on pro-inflammatory cytokine gene expression. It has been reported that immune system responses following radiation exposure differ across mouse strains and could be a factor when comparing our results to previous studies [67]. Furthermore, alterations to the microbiota we observe directly following spaceflight may have the ability to initiate an inflammatory state and alter TLR expression if followed for an extended period after return to Earth.

We observed a significantly higher relative expression of TGFβ in flight mice compared to ground controls, which contributes to epithelial migration and wound healing [68]. Previous studies have demonstrated that HLU can affect the ability to fight bacterial infection, and is thought to be due to impaired barrier integrity of the gastrointestinal tract [55, 66, 69]; however, immunohistological assessment of distal colon injury in flight animals does not indicate short-term space flight significantly affects epithelial integrity.

## Conclusion

To our knowledge, this is the first study to characterize the effect of both low and high LET radiation exposure, high dietary iron, and gravitational changes on the intestinal microbiota and colonic mucosal gene expression. A low dose, fractionated low LET radiation exposure or one low dose of high LET was chosen (for Experiments 1 and 2, respectively) to directly compare to the radiation exposure that astronauts experience during short-duration space flight missions; however, observations from these experiments could also provide insight for airline crews, radiation workers, and patients receiving heavy-ion cancer therapy. In Experiment 1 and 3, we did not observe a marked dysbiosis to the predominant phyla, reduced bacterial diversity, nor an increased proportion of pathogenic genera previously reported during intestinal inflammation and exposure to large doses of low LET radiation. Although minimal differences were observed in our selected gene targets, we have illustrated unique changes to the microbiota following elevated dietary iron consumption, radiation exposure (i.e., low LET and mixed radiation exposure), and space flight. Furthermore, we observed distinct shifts in bacterial populations in animals exposed to microgravity. These observations suggest unique alterations to the microbiota when exposed to a space environment compared to other disruptions to intestinal homeostasis such as gastrointestinal disease. Future studies analyzing alterations to the intestinal bacterial population’s genetic code, or metagenome, in patients receiving radiation therapy and astronauts would further elucidate how radiation exposure could affect intestinal homeostasis.

## Supporting Information

S1 Appendix Methodologies and results for Experiment 2. (DOCX)

S1 Table Assay ID for selected gene targets and relative expression in scraped colonic mucosa from mice exposed to radiation and reduced gravity (Experiment 2 and 3).^1^
(DOCX)

S2 Table Relative abundance (%) of bacterial taxa in feces of rats resulting from low LET radiation exposure and elevated dietary iron content (Experiment 1).^1^
(DOCX)

S3 Table Relative abundance (%) of bacterial taxa in feces of mice resulting from high LET radiation exposure and simulated lunar gravity (Experiment 2).^†^
(DOCX)

S4 Table Relative abundance (%) of bacterial taxa in feces of mice resulting from 13 days spaceflight (Experiment 3).^1^
(DOCX)
