# Supplementary material for: Space Environmental Factor Impacts upon Murine Colon Microbiota and Mucosal Homeostasis
Source: PLoS One. 2015 Jun 17;10(6):e0125792. doi: 10.1371/journal.pone.0125792 (PMC4470690; doi:10.1371/journal.pone.0125792)
Supplement: S1 Appendix — (DOCX) [file pone.0125792.s001.docx]

S1 Appendix. Methodologies and results for Experiment 2.

Materials and Methods

**Animals and diets**

*Experiment 2* - *high LET ^28^silicon particle exposure and partial weight bearing (1/6 G) in mice*

The aims of Experiment 2 were to determine the effect of high LET radiation and partial/lunar gravity (1/6 G) on: 1) fecal bacterial populations, and 2) expression of genes involved in microbial signaling and tissue repair.

Twenty-eight, 4-month-old, female BalbC/ByJ mice were purchased from Jackson Laboratories (Bar Harbor, ME) and shipped to the NASA Space Radiation Laboratory at Brookhaven National Laboratory (BNL) animal care facility. The animals were acclimated to single housing conditions, and a standard diet (Harlan Teklad 8604) and water were available ad libitum for 14 days prior to radiation exposure. After acclimation, half of the animals were suspended horizontally in cages by a device with a tension spring that could be adjusted to maintain 1/6 G weight bearing (LUN) on all four limbs as described previously [[19](#_ENREF_19), [20](#_ENREF_20)]. The remaining animals served as full weight bearing (WB) controls. Half of the LUN and WB mice were exposed to one dose of high LET ionizing ^28^Si (0.050 Gy at 300 MeV/nucleon, 0.25 Gy/min), simulating GCR. All animals were anesthetized with a ketamine:xylazine cocktail before euthanasia by decapitation on day 21. The colon was resected, feces removed, and the colonic mucosa scraped as part of a tissue sharing program.

**Sample collection and processing**

**Scraped mucosa**

After fecal material was removed, the colon was washed twice in RNase free Phosphate-Buffered Saline (PBS) and scraped on a chilled RNase free surface. Mucosa was transferred to an RNase free homogenization tube along with 500 μl of Denaturation Solution (Ambion, Austin, TX), homogenized by pipetting 6-7 times, snap frozen, then transported to Texas A&M University on dry ice and stored at -80°C.

*Feces collection and microbial DNA isolation*

Because of sample and funding limitations in this experiment, an aliquot of extracted bacterial DNA was taken from each animal and combined into a composite sample for each experimental group. The isolated composite DNA samples were used for pyrosequencing and further phylogenetic analysis in order to perform an initial and exploratory characterization of the impact of high LET radiation and unloading on microbiota.

**Measurement of gene expression using real-time PCR**

A complete description is provided in the manuscript.

**16S rRNA bacterial tag-encoded FLX amplicon pyrosequencing**

A complete description is provided in the manuscript.

**Amplicon sequencing data analysis**

A complete description is provided in the manuscript.

**Statistical analysis**

A complete description is provided in the manuscript.

**Results**

**Experiment 2 - Effect of high LET ^28^Si particle exposure and PWB on mucosal gene expression and the intestinal microbiota in mice**

**Microbial diversity, species richness and taxonomic structure analysis**

In an attempt to perform an initial and exploratory characterization of the impact of high LET radiation and reduced gravity on colon microbiota, we conducted an exploratory study in which composite samples of extracted microbial DNA were prepared for each experimental group. RAD numerically increased species richness in both WB and LUN groups (27% and 21%, respectively) with the greatest richness observed in LUN RAD mice (richness score 90.94). Compared to WB SHAM, we observed an increased abundance of *Bacteroidetes* and decreased abundance of *Firmicutes* in WB RAD (Supplemental Table 3). When comparing SHAM and RAD groups within each environmental treatment (WB vs LUN), we observed differential effects of radiation exposure on the characterized phyla. RAD increased *Bacteroidetes* and decreased *Firmicutes,* Unknown, Unassignable in WB, yet the inverse was observed in LUN RAD mice (Supplemental Table 3). In WB mice (SHAM and RAD groups) we observed a higher abundance of *Lactobacillales* (38-40%) and lower abundance of *Clostridiales* (10-13%). In contrast, LUN mice (SHAM and RAD groups) had higher abundance of *Clostridiales* (33-38%) and lower abundance of *Lactobacillales* (3-7%) (Supplemental Table 3).

**Mucosal gene expression**

We did not observe a significant effect of radiation or partial weight-bearing on relative expression of any gene targets analyzed (Supplemental Table 1). However, we observed increased relative expression of IL-1b in the LUN SHAM group relative to the WB SHAM mice. Similarly, expression of TLR4, MyD88, RelA/p65 (regulatory element for NF-κB), TFF3, and Slc5a8 tended to increase in the LUN RAD group compared to WB mice, especially the WB RAD mice.
