# Supplementary material for: Space Environmental Factor Impacts upon Murine Colon Microbiota and Mucosal Homeostasis
Source: PLoS One. 2015 Jun 17;10(6):e0125792. doi: 10.1371/journal.pone.0125792 (PMC4470690; doi:10.1371/journal.pone.0125792)
Supplement: S1 Table — (DOCX) [file pone.0125792.s002.docx]

S1 Table. Assay ID for selected gene targets and relative expression in scraped colonic mucosa from mice exposed to radiation and reduced gravity (Experiment 2 and 3).^1^

^^
