# Supplementary material for: Space Environmental Factor Impacts upon Murine Colon Microbiota and Mucosal Homeostasis
Source: PLoS One. 2015 Jun 17;10(6):e0125792. doi: 10.1371/journal.pone.0125792 (PMC4470690; doi:10.1371/journal.pone.0125792)
Supplement: S3 Table — (DOCX) [file pone.0125792.s004.docx]

S3 Table. Relative abundance (%) of bacterial taxa in feces of mice resulting from high LET radiation exposure and simulated lunar gravity (Experiment 2).^†^
