# Supplementary material for: Space Environmental Factor Impacts upon Murine Colon Microbiota and Mucosal Homeostasis
Source: PLoS One. 2015 Jun 17;10(6):e0125792. doi: 10.1371/journal.pone.0125792 (PMC4470690; doi:10.1371/journal.pone.0125792)
Supplement: S4 Table — (DOCX) [file pone.0125792.s005.docx]

S4 Table. Relative abundance (%) of bacterial taxa in feces of mice resulting from 13 days spaceflight (Experiment 3).^1^
